# Healthy Work Environment Assessment Tool - Brazilian version: validity and reliability assessment

**DOI:** 10.1590/0034-7167-2025-0279

**Published:** 2026-08-21

**Authors:** Renata Cristina Gasparino, Letícia Bianchini de Barros Minatogawa, Sharlla Milênia Nogueira da Silva, Letícia dos Santos Ribeiro, Olga Maria Pimenta Lopes Ribeiro, Andrea Bernardes

**Affiliations:** I Universidade de São Paulo. Ribeirão Preto, São Paulo, Brazil; II Universidade Estadual de Campinas. Campinas, São Paulo, Brazil; III Pontificia Universidad Católica de Valparaíso. Valparaíso, Chile; IV Escola Superior de Enfermagem do Porto. Porto, Portugal

**Keywords:** Reproducibility of Results, Health Facility Environment, Working Conditions, Health Care Professionals, Patient Safety., Reproducibilidad de los Resultados, Ambiente de Instituciones de Salud, Condiciones de Trabajo, Profesionales de la Salud, Seguridad del Paciente.

## Abstract

**Objectives:**

to assess the validity and reliability of the Brazilian version of the Healthy Work Environment Assessment Tool.

**Methods:**

a methodological study conducted with 145 healthcare professionals from a hospital in the countryside of São Paulo. For data collection, a sample characterization form, the Brazilian version of the Healthy Work Environment Assessment Tool, and sections B, C, and F of the Hospital Survey on Patient Safety Culture - Brazilian version were used. Confirmatory factor analysis and hypothesis testing (Spearman’s correlation coefficient) were used to assess validity. To assess reliability, composite reliability was calculated.

**Results:**

the six-factor structure with three items was confirmed. The correlation coefficient reached p<0.0001 for all hypotheses tested. Composite reliability ranged from 0.81 to 0.85.

**Conclusions:**

the Brazilian version of the tool showed evidence of validity and reliability, and is suitable for practical use.

## RESUMEN

**Objetivos:**

evaluar la validez y confiabilidad de la versión brasileña de la Healthy Work Environment Assessment Tool.

**Métodos:**

estudio metodológico realizado con 145 profesionales de la salud de un hospital del interior de São Paulo. Para la recolección de datos, se utilizó un formulario de caracterización de muestra, la versión brasileña de la Healthy Work Environment Assessment Tool y las secciones B, C y F de la Hospital Survey on Patient Safety Culture (versión brasileña). Se utilizaron análisis factorial confirmatorio y pruebas de hipótesis (coeficiente de correlación de Spearman) para evaluar la validez. Para evaluar la fiabilidad, se calculó la fiabilidad compuesta.

**Resultados:**

se confirmó la estructura de seis factores con tres ítems. El coeficiente de correlación alcanzó un valor de p<0,0001 para todas las hipótesis probadas. La fiabilidad compuesta osciló entre 0,81 y 0,85.

**Conclusiones:**

la versión brasileña del instrumento mostró evidencia de validez y fiabilidad, y es adecuada para su uso práctico.

## INTRODUCTION

The increased demand for healthcare professionals has been a global trend due to several factors, including an aging population, a rise in chronic diseases, and greater awareness of the importance of preventive healthcare^(1)^. Furthermore, the COVID-19 pandemic has brought even more prominence to the role of these professionals, since their work in dynamic and increasingly complex practice environments requires qualified and engaged teams that allow for efficient work^(2)^.

To ensure safe and high-quality care in the face of the most varied situations that arise, it is essential that policies be implemented to guarantee a dignified work environment for all professionals. This statement is supported by the World Health Organization, through various documents that highlight the importance of implementing a healthy work environment to achieve better healthcare outcomes^(3,4)^.

When addressing the construct of a healthy work environment, the American Association of Critical-Care Nurses (AACN) establishes six standards-skilled communication, true collaboration, effective decision-making, appropriate staffing, meaningful recognition, and authentic leadership-, which are imperative to ensure the achievement of better outcomes^(5,6)^.

Among these outcomes, we can mention the relationship between healthy work environments and psychological health, job satisfaction and retention, as well as a better perception of the institution’s care for professionals’ health and safety. With regard to patients, there are reports of decreased mortality rates and risk of complications, as well as increased satisfaction with the care received^(7,8)^. All these benefits, consequently, contribute to maintaining the organizations’ financial viability^(5,6)^.

Considering the above, it is essential that the work environment of healthcare professionals be rethought. The International Labour Organization also reinforces this cause by stating that decent work is not only an objective, but also an impetus for sustainable development, as it is the driving force behind economic growth^(9,10)^.

In order to make this transformation of work environments feasible, it is imperative that managers and researchers conduct studies that map opportunities for improvement and, consequently, help prioritize intervention strategies. To make this mapping feasible, valid and reliable tools that measure the patterns of healthy work environments are fundamental^(5)^.

To meet this demand, the AACN developed the Healthy Work Environment Assessment Tool (HWEAT) to measure the condition and progress of institutions in implementing and monitoring a healthy work environment^(5,6)^. Validated by a multidisciplinary team, HWEAT is one of the most widely used tools for this purpose^(5,7)^.

The application of HWEAT in Brazilian culture could demonstrate institutions’ commitment to the early recognition of behaviors that hinder the implementation of recommended standards, contributing to the review of processes carried out by the multidisciplinary team. This will help minimize/eliminate the occurrence of harm, promoting excellence in clinical practices and greater professional well-being^(5,6)^.

## OBJECTIVES

To assess the validity and reliability of HWEAT - Brazilian version.

## METHODS

### Ethical aspects

After obtaining consent from the author of the original instrument and approval from the institution where the study was conducted, the project was submitted to the *Universidade Estadual de Campinas* Research Ethics Committee, obtaining a favorable Opinion 5,523,598 (Certificate of Presentation for Ethical Consideration58525222.3.0000.5404).

### Study design, period, and location

This is a methodological study that sought to assess the construct validity and reliability of HWEAT - Brazilian version^(11)^, based on the COSMIN Reporting guideline for studies on measurement properties of patient-reported outcome measures^(12)^.

Data collection took place in person between April and June 2023 at a large university hospital in the countryside of São Paulo, SP, Brazil, which serves patients of the Brazilian Health System.

### Sample, inclusion and exclusion criteria

Considering the objective of assessing the tool’s measurement properties, the internationally recommended criterion was considered for the sample size calculation, being defined as “very good” when there are at least 100 participants and seven assessments per item of the instrument. Given that the tool has 18 items, the minimum calculated sample size was 126 healthcare professionals^(12)^. Participants were selected by convenience sampling at the hospital where the research was conducted. Eligible participants were nursing professionals, physicians, physiotherapists, nutritionists, pharmacists, and pharmacy technicians who had at least three months of experience at the institution. Since there were no blank responses, no participant was excluded from the study.

### Study protocol

The healthcare professionals on the multidisciplinary team were approached at their workplaces and during different shifts (morning, afternoon, and evening), and informed about the study’s topic and objectives, as well as ethical considerations. Participants who consented to participate in the study signed the Informed Consent Form and received printed questionnaires. The responses were submitted to the researchers on a pre-arranged date.

The instruments used for data collection were a sample profile description form, HWEAT - Brazilian version^(11)^ and sections B, C and F of the Hospital Survey on Patient Safety Culture (HSCOPSC) - Brazilian version^(13)^.

### Personal and professional profile sheet

It was developed with the aim of obtaining personal and professional information from the sample, such as sex, age, position, length of education and work experience in the organization, sector and training.

### Healthy Work Environment Assessment Tool - Brazilian version

HWEAT was adapted and its content validated within Brazilian culture^(11)^. It consists of a questionnaire composed of 18 items, divided into six patterns, with three items in each: A) Skilled communication (1, 6 and 14); B) True collaboration (2, 10 and 15); C) Effective decision-making (7, 11 and 16); D) Appropriate staffing (3, 8 and 12); E) Meaningful recognition (4, 9 and 17); and F) Authentic leadership (5, 13 and 18). A Likert scale is used to obtain the answers, where a score of 1 represents “strongly disagree” and a score of 5 represents “strongly agree”, i.e., higher scores indicate a greater perception of healthy characteristics in the work environment.

The final score is obtained by calculating the average of the responses for each domain, as well as the overall average for all items. From this calculation, the results can be classified as “need improvement” (1.00 - 2.99), “good” (3.00 - 3.99), and “excellent” (4.00 - 5.00)^(5)^.

In its original version, the tool’s Cronbach’s alpha coefficients ranged from 0.77 to 0.81. Regarding construct validity, factor analysis confirmed the initial distribution structure of items, with factor loadings ranging from 0.63 to 0.82. In hypothesis testing, the domains of the instrument in question demonstrated moderate to strong correlations with a section of HSOPSC^(5)^.

### Hospital Survey on Patient Safety Culture - Brazilian version

HSOPSC was developed to assess participants’ opinions on different dimensions that comprise central elements of the institution’s safety culture through 45 items, distributed across eight sections. In the present study, 21 items were used, which relate to the characteristics of healthy work environments, with four questions from section B (Your supervisor/boss), six questions from section C (Communication), and 11 questions from section F (Your hospital).

The items are assessed using a Likert scale, where the response options reflect participants’ level of agreement with the question. Scoring ranges from 1 (strongly disagree or never) to 5 (strongly agree or always), with a neutral category (3 - neither agree nor disagree or sometimes)^(13)^.

### Analysis of results

Data were entered into Microsoft Excel for Windows^®^ spreadsheets and processed using Statistical Analysis Software^®^ version 9.4 and Smart PLS^®^ software version4.0.9.0. Categorical variables were descriptively analyzed, and the results are presented as absolute and relative frequencies. For continuous variables, measures of position and dispersion were calculated. The scores for the instruments’ patterns/sections used were obtained by averaging the responses provided by the participating professionals.

To verify construct validity, the tool’s structure was analyzed using confirmatory factor analysis, where the environment was considered a second-order variable, employing structural equation modeling and adopting the Partial Least Squares (PLS) estimation method. The factor model assessment was performed in two stages: convergent validity analysis and discriminant validity analysis of the developed model.

Firstly, the values of the average variance extracted (AVE), which assesses the extent to which the factor is represented by its component items, were determined, and those above 0.5 demonstrate that the model shows results with evidence of convergence^(14)^.

Furthermore, the Fornell-Larcker criterion and cross-loadings were used to assess the model’s discriminant validity. Concerning cross-loadings, the goal was for the item’s factor loading to be higher in the standard to which it was originally assigned. For the Fornell-Larcker criterion, the expectation was that the square roots of the item’s AVE between the same constructs would be higher than the correlations between the standards. Internal consistency was used to assess reliability, using Cronbach’s alpha coefficient and composite reliability (CR), with values ≥0.60 and ≥0.70 considered satisfactory, respectively^(14)^.

Regarding construct validity, a hypothesis test was applied, formulating the hypothesis that the higher the score on the standards of HWEAT - Brazilian version, the higher the score on sections B, C, and F of HSOPSC - Brazilian version. Spearman’s correlation coefficient was used to assess data distribution. P-values <0.05 were considered significant.

## RESULTS

The sample consisted of 145 professionals from the multidisciplinary team. The majority of participants were female (75.86%; n=110), with a mean age of 37.77 ± 10.47 years. Among them, 20 professionals (13.79%) formed a group composed of pharmacists, pharmacy technicians, and nutritionists. In addition, 50 were physicians (34.48%), 23 were nurses (15.86%), and 52 were nursing technicians (35.86%). On average, they had graduated 11.03 ± 9.92 years ago and had 6.93 ± 8.30 years of experience at the institution. Most worked in inpatient units (60%; n=87). Regarding education, 63 (43.45%) had completed *lato sensu* graduate courses.

For structure assessment, the AVE, CR, and Cronbach’s alpha of each of the tool’s patterns were determined, with the results presented in Table 1.

**Table 1:** Average variance extracted, composite reliability, and Cronbach’s alpha of the standards from the Brazilian version of the Healthy Work Environment Assessment Tool (N=145), Campinas, São Paulo, Brazil, 2023

Healthy Work Environment Assessment Tool - Brazilian version domains	Average variance extracted	Composite reliability	Cronbach’s alpha
True collaboration	0.61	0.82	0.67
Effective decision-making	0.64	0.84	0.72
Appropriate staffing	0.62	0.83	0.69
Skilled communication	0.59	0.81	0.66
Authentic leadership	0.66	0.85	0.75
Meaningful recognition	0.63	0.83	0.70

To assess the model’s discriminant validity, the item loadings for each of the factors were presented in Table 2 so that the cross-factor loadings could be analyzed

**Table 2:** Factor loadings of the items in the Healthy Work Environment Assessment Tool - Brazilian version (N=145), Campinas, São Paulo, Brazil, 2023

	True collaboration	Effective decision-making	Appropriate staffing	Skilled communication	Authentic leadership	Meaningful recognition
						
Q1	0.60	0.60	0.60	0.81	0.53	0.57
Q2	0.76	0.50	0.46	0.61	0.39	0.49
Q3	0.48	0.52	0.80	0.48	0.56	0.53
Q4	0.50	0.46	0.57	0.51	0.55	0.83
Q5	0.38	0.39	0.50	0.48	0.80	0.51
Q6	0.46	0.45	0.41	0.80	0.46	0.46
Q7	0.56	0.86	0.64	0.57	0.54	0.58
Q8	0.48	0.60	0.82	0.50	0.62	0.59
Q9	0.49	0.47	0.44	0.47	0.47	0.76
Q10	0.73	0.50	0.44	0.44	0.48	0.50
Q11	0.52	0.79	0.60	0.49	0.47	0.44
Q12	0.47	0.55	0.73	0.49	0.41	0.43
Q13	0.61	0.60	0.63	0.57	0.86	0.61
Q14	0.47	0.44	0.41	0.70	0.44	0.46
Q15	0.84	0.51	0.52	0.51	0.56	0.50
Q16	0.47	0.75	0.46	0.51	0.46	0.43
Q17	0.52	0.50	0.56	0.55	0.52	0.79
Q18	0.48	0.48	0.52	0.46	0.77	0.47
						

Table 3 presents the AVE square root and the correlations between the patterns of the Brazilian version of the tool so that the Fornell-Larcker criterion can be analyzed.

**Table 3:** Square root mean variance extracted and correlations between the patterns of the Healthy Work Environment Assessment Tool - Brazilian version (N=145), Campinas, São Paulo, Brazil, 2023

	True collaboration	Effective decision-making	Appropriate staffing	Skilled communication	Authentic leadership	Meaningful recognition
						
**True collaboration**	0.78					
**Effective decision-making**	0.65	0.80				
**Appropriate staffing**	0.61	0.71	0.79			
**Skilled communication**	0.67	0.65	0.63	0.77		
**Authentic leadership**	0.61	0.61	0.69	0.62	0.81	
**Meaningful recognition**	0.64	0.61	0.66	0.65	0.66	0.79
						

To assess construct validity through hypothesis testing, correlations were tested between each HWEAT pattern and sections B, C, and F of HSOPSC - Brazilian version (Table 4).

**Table 4:** Correlations between the patterns of the Brazilian version of the Healthy Work Environment Assessment Tool and sections B, C, and F of the Brazilian version of the Hospital Survey on Patient Safety Culture (N=145), Campinas, São Paulo, Brazil, 2023

	Skilled communication	True collaboration	Effective decision-making	Appropriate staffing	Meaningful recognition	Authentic leadership
**HSOPSC - B**	0.4688	0.4313	0.4375	0.4224	0.4881	0.4997
	<0.0001^*^	<0.0001^*^	<0.0001^*^	<0.0001^*^	<0.0001^*^	<0.0001^*^
**HSOPSC - C**	0.5582	0.4810	0.5415	0.5267	0.5879	0.5359
	<0.0001^*^	<0.0001^*^	<0.0001^*^	<0.0001^*^	<0.0001^*^	<0.0001^*^
**HSOPSC - F**	0.5169	0.4214	0.4898	0.5827	0.4533	0.4970
	<0.0001^*^	<0.0001^*^	<0.0001^*^	<0.0001^*^	<0.0001^*^	<0.0001^*^

HSOPSC - Hospital Survey on Patient Safety Culture;

* Statistical test: Spearman’s correlation.

When the domain scores and the instrument total score were assessed, it was observed that the lowest score was obtained in the “Meaningful recognition” domain (2.81 ± 0.82), while the highest score was found in the “Authentic leadership” domain (3.45 ± 0.78). Regarding the total number of items in the tool, the score obtained was 3.17 ± 0.67.

## DISCUSSION

The use of rigorously validated instruments is essential to ensure reliable and safe tools in both clinical practice and research. The literature indicates that the properties of an instrument can be measured through its reliability, validity, and responsiveness^(12)^. In this study, different measures were adopted because these approaches are understood to be both independent and complementary.

To this end, a sample of participants from the multidisciplinary team was formed. Data analysis revealed that pharmacists, pharmacy technicians, and nutritionists constituted the smallest portion of the sample, as the researchers respected the proportion of professionals in the institution. A similar result was found in the validity study of the original version of the tool, in which, disregarding blank responses, physicians, and nursing staff, approximately 14% of the team consisted of other healthcare professionals^(5)^.

The validity of an instrument refers to its ability to accurately measure the intended construct, and, in this study, it was verified by assessing the tool’s structure (confirmatory factor analysis) and by hypothesis testing. When comparing the analysis performed in the present study with that performed in Canadian and Japanese cultures^(15,16)^, it was noted that all researchers opted for confirmatory analysis because they considered that the six-factor structure had already been tested in the original version^(5)^. However, unlike the validity for Brazilian culture, none of the three studies cited employed structural equation modeling, using PLS as an estimation method in their analyses. This method is recommended when researchers intend to estimate complex models with a smaller amount of data^(14)^.

As a result of factor analysis, the structure of the original version of HWEAT was confirmed, as the items in the Brazilian version were distributed in the same way and proved adequate to the six patterns. AVEs explained more than 50% of constructs, indicating that the items measure the constructs they are intended to measure^(14)^.

The factor loadings of items ranged from 0.70 to 0.86, demonstrating the importance of the items for the construct being measured^(14)^. These values were higher than those obtained in the research that validated the initial version of the tool (0.63 - 0.82)^(5)^ and in the tool validity for Japanese culture (0.63 - 0.82)^(16)^.

Upon examining the cross-loadings, it was found that the items were appropriately allocated within the domains, demonstrating that the model possesses discriminant validity insofar as the square roots of AVEs between the same constructs were greater than the correlations between the other factors^(14)^.

Regarding internal consistency, it was noted that the Cronbach’s alpha coefficients achieved in the present study were lower than those found in other previously published studies^(5,16-18)^. However, considering what is recommended by the literature^(14)^, the data obtained demonstrated evidence of reliability. It is worth highlighting that the present research also used CC to measure reliability, as it is considered a more appropriate measure due to its lower sensitivity with respect to the number of items in each construct^(14)^.

In addition to factor analysis, another way to assess construct validity was through hypothesis testing. The initially formulated hypothesis was confirmed, as significant relationships of moderate to strong magnitudes were found between the patterns of HWEAT - Brazilian version and the three sections of HSOPSC - Brazilian version. These results demonstrate that a healthy work environment contributes positively to the consolidation of a safer patient culture, as also observed by other authors^(19)^.

Hypothesis testing was also used to validate the original version of the tool. A section of HSOPSC was used for this purpose, and results similar to those of the Brazilian version were found^(5)^. The hypothesis test of the Canadian version was conducted considering one question about nurses’ perception of quality of care and another about care safety. The results showed moderate positive correlations, indicating that healthy work environments also contribute to quality of care^(15)^.

Taking into account the studies that aimed to validate the instrument^(5,15,16)^, it was noted that in the United States (3.58 points) and Saudi Arabia (3.64 points), professionals’ perception was more positive than that of the professionals in the present study^(5,17)^. However, in the Canadian (2.96 points) and Japanese (3.00 points) versions, professionals demonstrated a more negative perception when compared to Brazilians^(15,16)^. These results classified the environments as “good” and “in need of improvement”, demonstrating the possibility for institutions to further explore and implement strategies to improve professionals’ working conditions.

Regarding the patterns, the data from the Brazilian version were similar to those of other studies, especially with respect to the meaningful recognition pattern, which has received the lowest score in research^(5,16-18,20)^. Data from a longitudinal study assessing the implementation of standards for a healthy work environment showed that both before and after the intervention, meaningful recognition received the worst rating from professionals^(21)^. Recognition is paramount and forms the core of standards, as its absence can contribute to people developing feelings of invisibility, devaluation, demotivation, disrespect, and dissatisfaction^(6)^. Therefore, informing professionals about the excellence of their performance reinforces their value in providing quality patient care^(17)^.

The authors reinforce several measures that can be implemented to enhance meaningful recognition, in addition to strategies that involve increased costs, such as greater flexibility in working hours, encouragement of professional development, and awards for professionals nominated by patients and colleagues. Recognition should become a priority for leadership, and each achievement should be publicly celebrated^(22)^.

Implementing these standards, including behavioral ones, should be a priority for healthcare organizations, as it allows for workforce stability, the promotion of a safer culture, improved performance and productivity of professionals, and improved quality of patient care and financial viability of institutions^(7,17,23)^. Therefore, it is recommended by the United Nations, the World Health Organization, and the International Labour Organization as a driver of sustainable development^(4,9,10)^.

### Study limitations

Limitations of this research include the convenience sampling method, which may lead to selection bias, and the use of a self-administered tool, which may be subject to social desirability bias. The lack of other instruments to measure outcomes with professionals, such as job satisfaction or occupational stress, and conducting the research in a single setting, which may not represent the entirety of the culture considering the vastness of Brazil, can also be cited as limitations.

### Contributions to health

The availability of HWEAT - Brazilian version will enable the mapping and subsequent assessment of institutions’ progress in implementing and consolidating a healthy work environment. This tool can assist managers in the efforts and investments necessary to implement training programs, a culture of recognition, zero-tolerance policies regarding abusive and disrespectful behavior, as well as combat the traditional gap in interprofessional cooperation and understand that adequately meeting patients’ needs requires a greater number of qualified professionals^(6)^.

Since they are not considered stable^(12)^, the following actions could be taken in order to better enable the generalization of results: future research carried out in several institutions and taking into account a probabilistic sampling process; the inclusion of new measurement instruments that measure the relationship between healthy work environments and the outcomes observed in patients, teams and organizations; the mapping of characteristics of environments in different regions and Brazilian scenarios that allow benchmarking; the carrying out of longitudinal studies that monitor the progress of the implementation of strategies aimed at fostering a healthy work environment^(5,6)^; and confirmation of the instrument’s measurement properties.

## CONCLUSIONS

HWEAT - Brazilian version presented evidence of validity and reliability, allowing its use with multidisciplinary teams working in hospital settings. The tool maintained an identical structure to the original version (18 items distributed across six patterns), and hypothesis testing demonstrated that a healthy work environment contributes to improving patient safety culture.

## Data Availability

The research data are available in a repository: https://doi.org/10.25824/redu/Q9Q0FM.
