# Supplementary material for: Healthy Work Environment Assessment Tool - Brazilian version: validity and reliability assessment
Source: Rev Bras Enferm. 2026 Aug 21;79:e20250279. doi: 10.1590/0034-7167-2025-0279 (PMC13506214; doi:10.1590/0034-7167-2025-0279)
Supplement: Translation

# Translation: *Healthy Work Environment Assessment Tool* - versão brasileira: avaliação da validade e da confiabilidade

**DOI:** 10.1590/0034-7167-2025-0279pt

**Authors:** Renata Cristina Gasparino, Letícia Bianchini de Barros Minatogawa, Sharlla Milênia Nogueira da Silva, Letícia dos Santos Ribeiro, Olga Maria Pimenta Lopes Ribeiro, Andrea Bernardes

**Affiliations:** I Universidade de São Paulo. Ribeirão Preto, São Paulo, Brasil; II Universidade Estadual de Campinas. Campinas, São Paulo, Brasil; III Pontificia Universidad Católica de Valparaíso. Valparaíso, Chile; IV Escola Superior de Enfermagem do Porto. Porto, Portugal

**Keywords:** Reprodutibilidade dos Testes, Ambiente de Instituições de Saúde, Condições de Trabalho, Profissionais de Saúde, Segurança do Paciente.

## Abstract

**Objetivos:**

avaliar a validade e a confiabilidade da versão brasileira da *Healthy Work Environment Assessment Tool*.

**Métodos:**

estudo metodológico, realizado com 145 profissionais de saúde de hospital do interior de São Paulo. Para a coleta de dados, foram utilizadas uma ficha para caracterização da amostra, a versão brasileira da *Healthy Work Environment Assessment Tool* e as seções B, C e F da *Hospital Survey on Patient Safety Culture* - versão brasileira. Para avaliação da validade, utilizaram-se a análise fatorial confirmatória e o teste de hipóteses (coeficiente de correlação de Spearman). Para avaliação da confiabilidade, calculou-se a confiabilidade composta.

**Resultados:**

a estrutura de seis fatores com três itens foi confirmada. O coeficiente de correlação alcançou p<0,0001 para todas as hipóteses testadas. A confiabilidade composta variou entre 0,81 e 0,85.

**Conclusões:**

a versão brasileira da ferramenta apresentou evidências de validade e confiabilidade, estando apta para ser utilizada na prática.

## INTRODUÇÃO

O aumento da demanda por profissionais de saúde tem sido uma tendência global devido a diversos fatores, incluindo o envelhecimento da população, o aumento das doenças crônicas e a maior conscientização sobre a importância dos cuidados de saúde preventivos^(1)^. Além disso, a pandemia de COVID-19 trouxe ainda mais destaque para o papel desses profissionais, uma vez que a atuação dos mesmos em ambientes de práticas dinâmicos e cada vez mais complexos exige equipes qualificadas e engajadas que permitam um trabalho eficiente^(2)^.

Para se garantir um cuidado seguro e de qualidade diante das mais variadas situações que se apresentam, é essencial que políticas sejam implementadas com o intuito de garantir um ambiente de trabalho digno para todos os profissionais. Essa afirmação é respaldada pela Organização Mundial da Saúde, por meio de diferentes documentos que evidenciam a relevância da implementação de um ambiente de trabalho saudável para a consecução de melhores resultados assistenciais^(3,4)^.

Ao se abordar o construto ambiente de trabalho saudável, a *American Association of Critical-Care Nurses* (AACN) estabelece seis padrões-comunicação, colaboração verdadeira, efetiva tomada de decisões, equipe apropriada, reconhecimento significativo e liderança autêntica-, que são imperativos para se assegurar o alcance de melhores desfechos^(5,6)^.

Entre esses desfechos, pode ser citada a relação entre ambientes de trabalho saudáveis e saúde psicológica, satisfação e retenção profissional, além de melhor percepção do cuidado da instituição com a saúde e a segurança dos profissionais. Com relação ao paciente, encontram-se relatos sobre a diminuição das taxas de mortalidade e risco de complicações, além de aumento da satisfação com os cuidados recebidos^(7,8)^. Todos esses benefícios, consequentemente, contribuem para a manutenção da viabilidade financeira das organizações^(5,6)^.

Considerando o exposto, revela-se essencial que o ambiente de trabalho dos profissionais de saúde seja repensado. A Organização Internacional do Trabalho também reforça essa causa, ao afirmar que o trabalho digno não é apenas um objetivo, mas um impulso ao desenvolvimento sustentável, pois é o propulsionador do crescimento econômico^(9,10)^.

Com o intuito de tornar factível essa transformação dos ambientes de trabalho, é imperioso que gestores e pesquisadores conduzam estudos que mapeiem as oportunidades de melhoria e, consequentemente, auxiliem na priorização de estratégias de intervenção. Para tornar esse mapeamento factível, ferramentas válidas e confiáveis, que mensurem os padrões de ambientes de trabalho saudáveis, são fundamentais^(5)^.

Para atender a essa demanda, a AACN desenvolveu a *Healthy Work Environment Assessment Tool* (HWEAT) com o propósito de mensurar a condição e o avanço das instituições na implementação e no monitoramento de um ambiente de trabalho saudável^(5,6)^. Validada com uma equipe multiprofissional, a HWEAT é um dos instrumentos mais utilizados para esta finalidade^(5,7)^.

A aplicação da HWEAT na cultura brasileira poderá demonstrar o compromisso das instituições no reconhecimento precoce de comportamentos que dificultem a implementação dos padrões recomendados, contribuindo para a revisão dos processos realizados pela equipe multiprofissional, que auxiliarão na minimização/eliminação da ocorrência de danos, promovendo excelência das práticas clínicas e maior bem-estar profissional^(5,6)^.

## OBJETIVOS

Avaliar a validade e a confiabilidade da versão brasileira da HWEAT.

## MÉTODOS

### Aspectos éticos

Após a obtenção do consentimento do autor do instrumento original e da aprovação pela instituição onde o estudo foi conduzido, o projeto foi submetido ao Comitê de Ética em Pesquisa da Universidade Estadual de Campinas, obtendo Parecer favorável nº 5.523.598 (Certificado de Apresentação para Apreciação Ética nº 58525222.3.0000.5404).

### Desenho, período e local do estudo

Trata-se de estudo metodológico, que buscou avaliar a validade de construto e a confiabilidade da versão brasileira da HWEAT^(11)^, tendo como base as diretrizes do *COSMIN Reporting guideline for studies on measurement properties of patient-reported outcome measures* ^(12)^.

A coleta de dados ocorreu presencialmente entre abril e junho de 2023, em um hospital universitário de grande porte no interior de São Paulo, SP, Brasil, que atende pacientes do Sistema Único de Saúde.

### Amostra, critérios de inclusão e exclusão

Tendo em vista o objetivo de avaliar as propriedades de medida da ferramenta, para o cálculo amostral, foi considerado o critério internacionalmente recomendado, sendo “muito bom” quando há, no mínimo, 100 participantes e sete avaliações por item do instrumento. Considerando que a ferramenta possui 18 itens, a amostra mínima calculada foi de 126 profissionais de saúde^(12)^. Os participantes foram selecionados por conveniência no hospital em que a pesquisa foi realizada, sendo considerados aptos os profissionais de enfermagem, médicos, fisioterapeutas, nutricionistas, farmacêuticos e técnicos de farmácia que tivessem, no mínimo, três meses de experiência na instituição. Considerando que não houve respostas em branco, nenhum participante foi excluído do estudo.

### Protocolo do estudo

Os profissionais de saúde da equipe multidisciplinar foram abordados nos locais em que desenvolvem suas atividades e em diferentes turnos (manhã, tarde e noite), orientados quanto ao tema e objetivo do estudo, bem como aos aspectos éticos. Os participantes que consentiram integrar o estudo assinaram o Termo de Consentimento Livre e Esclarecido, e receberam os formulários impressos. As respostas foram entregues aos pesquisadores em uma data combinada.

Os instrumentos utilizados para a coleta de dados foram uma ficha para descrição do perfil amostral, a HWEAT - versão brasileira^(11)^ e as seções B, C e F da *Hospital Survey on Patient Safety Culture* (HSOPSC) - versão brasileira^(13)^.

### Ficha de caracterização pessoal e profissional

Foi elaborada com o intuito de obter informações pessoais e profissionais da amostra, como sexo, idade, cargo, tempo de formação e de atuação na organização, setor e formação.

### *Healthy Work Environment Assessment Tool* - versão brasileira

A HWEAT foi adaptada, e teve seu conteúdo validado na cultura brasileira^(11)^. Trata-se de questionário composto por 18 itens, divididos em seis padrões, com três itens em cada: A) Habilidades de comunicação (1, 6 e 14); B) Colaboração verdadeira (2, 10 e 15); C) Efetividade na tomada de decisões (7, 11 e 16); D) Equipe apropriada (3 ,8 e 12); E) Reconhecimento significativo (4, 9 e 17); e F) Liderança autêntica (5, 13 e 18). Para a obtenção das respostas, utiliza-se uma escala Likert, em que o escore 1 representa “discordo totalmente”, e o escore 5, “concordo totalmente”, isto é, pontuações mais altas indicam maior percepção de características saudáveis no ambiente de trabalho.

A pontuação final é obtida por meio do cálculo da média das respostas de cada domínio, bem como da média geral para todos os itens. A partir deste cálculo, os resultados podem ser classificados entre “necessitam de melhorias” (1,00 - 2,99), “bons” (3,00 - 3,99) e “excelentes” (4,00 - 5,00)^(5)^.

Em sua versão original, os coeficientes alfa de Cronbach da ferramenta variaram entre 0,77 e 0,81. Com relação à validade de construto, a análise fatorial confirmou a estrutura inicial de distribuição dos itens, com carga fatorial que variou entre 0,63 e 0,82. No teste de hipótese, os domínios do instrumento em questão demonstraram moderadas e fortes correlações com uma seção da HSOPSC^(5)^.

### *Hospital Survey on Patient Safety Culture* - versão brasileira

A HSOPSC foi elaborada com o intuito de avaliar a opinião dos participantes sobre diferentes dimensões que compõem elementos centrais da cultura de segurança da instituição por meio de 45 itens, distribuídos em oito seções. No presente estudo, foram utilizados 21 itens, que têm relação com as características de ambientes de trabalho saudáveis, sendo quatro questões da seção B (O seu supervisor/chefe), seis questões da seção C (Comunicação) e 11 questões da seção F (O seu hospital).

A avaliação dos itens é feita com uma escala Likert cujas opções de resposta refletem o nível de concordância do participante com relação ao que está sendo indagado. A variação da pontuação ocorre entre 1 (discordo totalmente ou nunca) e 5 (concordo totalmente ou sempre), com uma categoria neutra (3 - não concordo nem discordo ou às vezes)^(13)^.

### Análise dos resultados

Os dados foram inseridos em planilhas do *Microsoft Excel for Windows^®^* e processados pelo *Statistical Analysis Software^®^,* versão 9.4, e pelo *software SmartPLS^®^,* versão 4.0.9.0. As variáveis categóricas foram descritivamente analisadas, e os resultados, apresentados por frequência absoluta e relativa. Para as variáveis contínuas, foram calculadas as medidas de posição e dispersão. Os escores dos padrões/seções dos instrumentos utilizados foram obtidos por meio da média das respostas fornecidas pelos profissionais que participaram.

Com o objetivo de checar a validade de construto, foi realizada a análise da estrutura da ferramenta por meio de análise fatorial confirmatória, cujo ambiente foi considerado uma variável de segunda ordem, utilizando modelagem de equações estruturais e adotando o método de estimação *Partial Least Squares* (PLS). A avaliação do modelo fatorial foi realizada em duas etapas: análise da validade convergente e discriminante do modelo desenvolvido.

Primeiramente, os valores da variância média extraída (VME), que avalia em que medida o fator é representado pelos itens que o compõe, foram determinados, e aqueles acima de 0,5 demonstram que o modelo apresenta resultados com evidências de convergência^(14)^.

Além disso, para avaliação da validade discriminante do modelo, foram utilizados o critério de Fornell-Larcker e as cargas cruzadas. Quanto às cargas cruzadas, desejava-se que a carga fatorial do item fosse mais alta no padrão ao qual foi originalmente designado. Para Fornell-Larcker, aguardava-se que as raízes quadradas das VME entre os mesmos construtos fossem superiores do que as correlações entre os padrões. A consistência interna foi utilizada para avaliar a confiabilidade, por meio do coeficiente alfa de Cronbach e confiabilidade composta (CC), sendo valores ≥0,60 e ≥0,70 considerados satisfatórios, respectivamente^(14)^.

Em relação à validade de construto, foi aplicado o teste de hipóteses, em que a seguinte hipótese foi formulada: quanto maior o escore nos padrões da versão brasileira da HWEAT, maior a pontuação nas seções B, C e F da versão brasileira da HSOPSC. Para isso, utilizou-se o coeficiente de correlação de Spearman, em função da distribuição dos dados. Foram considerados significantes os valores de p<0,05.

## RESULTADOS

A amostra foi composta por 145 profissionais da equipe multidisciplinar. A maioria dos participantes era do sexo feminino (75,86%; n=110), com idade média de 37,77 ± 10,47 anos. Entre eles, 20 profissionais (13,79%) formaram um grupo composto por farmacêuticos, técnicos de farmácia e nutricionistas. Além disso, 50 eram médicos (34,48%), 23, enfermeiros (15,86%), e 52, técnicos de enfermagem (35,86%). Em média, haviam se formado há 11,03 ± 9,92 anos e possuíam 6,93 ± 8,30 anos de tempo de atuação na instituição. A maioria atuava em unidades de internação (60%; n=87). Com relação à formação, 63 (43,45%) possuíam pós-graduação *lato sensu* concluída.

Para a avaliação da estrutura, foram determinados a VME, a CC e o alfa de Cronbach de cada um dos padrões da ferramenta, sendo os resultados apresentados na Tabela 1.

**Tabela 1:** Variância média extraída, confiabilidade composta e alfa de Cronbach dos padrões da versão brasileira da *Healthy Work Environment Assessment Tool* (N=145), Campinas, São Paulo, Brasil, 2023

Domínios da versão brasileira da *Healthy Work Environment Assessment Tool*	Variância média extraída	Confiabilidade composta	Alfa de Cronbach
Colaboração verdadeira	0,61	0,82	0,67
Efetividade na tomada de decisões	0,64	0,84	0,72
Equipe apropriada	0,62	0,83	0,69
Habilidades de comunicação	0,59	0,81	0,66
Liderança autêntica	0,66	0,85	0,75
Reconhecimento significativo	0,63	0,83	0,70

Para avaliação da validade discriminante do modelo, as cargas dos itens referentes a cada um dos padrões foram apresentadas na Tabela 2, para que as cargas fatoriais cruzadas fossem analisadas.

**Tabela 2:** Cargas fatoriais dos itens da *Healthy Work Environment Assessment Tool* - versão brasileira (N=145), Campinas, São Paulo, Brasil, 2023

	Colaboração verdadeira	Efetividade na tomada de decisões	Equipe apropriada	Habilidades de comunicação	Liderança autêntica	Reconhecimento significativo
						
Q1	0,60	0,60	0,60	0,81	0,53	0,57
Q2	0,76	0,50	0,46	0,61	0,39	0,49
Q3	0,48	0,52	0,80	0,48	0,56	0,53
Q4	0,50	0,46	0,57	0,51	0,55	0,83
Q5	0,38	0,39	0,50	0,48	0,80	0,51
Q6	0,46	0,45	0,41	0,80	0,46	0,46
Q7	0,56	0,86	0,64	0,57	0,54	0,58
Q8	0,48	0,60	0,82	0,50	0,62	0,59
Q9	0,49	0,47	0,44	0,47	0,47	0,76
Q10	0,73	0,50	0,44	0,44	0,48	0,50
Q11	0,52	0,79	0,60	0,49	0,47	0,44
Q12	0,47	0,55	0,73	0,49	0,41	0,43
Q13	0,61	0,60	0,63	0,57	0,86	0,61
Q14	0,47	0,44	0,41	0,70	0,44	0,46
Q15	0,84	0,51	0,52	0,51	0,56	0,50
Q16	0,47	0,75	0,46	0,51	0,46	0,43
Q17	0,52	0,50	0,56	0,55	0,52	0,79
Q18	0,48	0,48	0,52	0,46	0,77	0,47
						

Na Tabela 3, foram apresentadas a raiz quadrada da VME e as correlações entre os padrões da versão brasileira da ferramenta, para que o critério de Fornell-Larcker pudesse ser analisado.

**Tabela 3:** Raiz quadrada da variância média extraída e correlações entre os padrões da *Healthy Work Environment Assessment Tool* - versão brasileira (N=145), Campinas, São Paulo, Brasil, 2023

	Colaboração verdadeira	Efetividade na tomada de decisões	Equipe apropriada	Habilidades de comunicação	Liderança autêntica	Reconhecimento significativo
						
**Colaboração verdadeira**	0,78					
**Efetividade na tomada de decisões**	0,65	0,80				
**Equipe apropriada**	0,61	0,71	0,79			
**Habilidades de comunicação**	0,67	0,65	0,63	0,77		
**Liderança autêntica**	0,61	0,61	0,69	0,62	0,81	
**Reconhecimento significativo**	0,64	0,61	0,66	0,65	0,66	0,79
						

Para avaliação da validade de construto por meio do teste de hipótese, foram testadas as correlações entre cada padrão da HWEAT, além das seções B, C e F da versão brasileira da HSOPSC (Tabela 4).

**Tabela 4:** Correlações entre os padrões da versão brasileira da *Healthy Work Environment Assessment Tool* e as seções B, C e F da versão brasileira da *Hospital Survey on Patient Safety Culture* (N=145), Campinas, São Paulo, Brasil, 2023

	Habilidades comunicação	Colaboração verdadeira	Efetividade na tomada de decisões	Equipe apropriada	Reconhecimento significativo	Liderança autêntica
**HSOPSC - B**	0,4688	0,4313	0,4375	0,4224	0,4881	0,4997
	<0,0001^*^	<0,0001^*^	<0,0001^*^	<0,0001^*^	<0,0001^*^	<0,0001^*^
**HSOPSC - C**	0,5582	0,4810	0,5415	0,5267	0,5879	0,5359
	<0,0001^*^	<0,0001^*^	<0,0001^*^	<0,0001^*^	<0,0001^*^	<0,0001^*^
**HSOPSC - F**	0,5169	0,4214	0,4898	0,5827	0,4533	0,4970
	<0,0001^*^	<0,0001^*^	<0,0001^*^	<0,0001^*^	<0,0001^*^	<0,0001^*^

HSOPSC - Hospital Survey on Patient Safety Culture;

* Teste estatístico: correlação de Spearman.

Quando os escores dos domínios e do total do instrumento foram avaliados, observou-se que o menor escore foi obtido no domínio “Reconhecimento significativo” (2,81 ± 0,82), enquanto o maior escore foi verificado no domínio “Liderança autêntica” (3,45 ± 0,78). Com relação ao total de itens da ferramenta, o escore obtido foi de 3,17 ± 0,67.

## DISCUSSÃO

A utilização de instrumentos validados com rigor metodológico é essencial para garantir ferramentas confiáveis e seguras tanto na prática clínica quanto na pesquisa. A literatura indica que as propriedades de um instrumento podem ser mensuradas por meio da confiabilidade, validade e responsividade^(12)^. No presente estudo, foram adotadas diferentes medidas por compreender que essas abordagens são independentes e complementares.

Para isso, uma amostra com participantes da equipe multiprofissional foi constituída. Na análise de dados, foi possível notar que os farmacêuticos, técnicos de farmácia e nutricionistas constituíram a menor parte da amostra, pois os pesquisadores respeitaram a proporção de profissionais da instituição. Resultado semelhante foi encontrado na pesquisa de validação da versão original da ferramenta, na qual, desconsiderando as respostas em branco, médicos e equipe de enfermagem, aproximadamente 14% da equipe era constituída pelos demais profissionais da área da saúde^(5)^.

A validade de um instrumento se refere à sua aptidão para mensurar de forma precisa o construto pretendido, e, neste estudo, foi verificada pela avaliação da estrutura da ferramenta (análise fatorial confirmatória) e pelo teste de hipótese. Ao comparar a análise realizada no presente estudo com a realizada nas culturas canadense e japonesa^(15,16)^, notou-se que todos os pesquisadores optaram pela análise confirmatória por considerarem que a estrutura de seis fatores já havia sido testada na versão original^(5)^. Entretanto, diferentemente da validação para a cultura brasileira, nenhum dos três estudos citados empregou modelagem de equações estruturais, utilizando o PLS como método de estimação em suas análises. Este método é recomendado quando os pesquisadores pretendem estimar modelos complexos com um menor número de dados^(14)^.

Como resultado da análise fatorial, a estrutura da versão original da HWEAT foi confirmada, pois os itens da versão brasileira se distribuíram da mesma forma, mostrando-se adequados aos seis padrões. As VME explicaram mais de 50% dos construtos, indicando que os itens mensuram os construtos aos quais se propõem^(14)^.

As cargas fatoriais dos itens variaram entre 0,70 e 0,86, demonstrando a importância dos itens para o construto que está sendo mensurado^(14)^. Esses valores foram superiores aos obtidos na pesquisa que validou a versão inicial da ferramenta (0,63 - 0,82)^(5)^ e na validação da ferramenta para a cultura japonesa (0,63 - 082)^(16)^.

Ao examinar as cargas cruzadas, verificou-se que os itens estavam adequadamente alocados nos domínios, demonstrando que o modelo possui validade discriminante, pois as raízes quadradas das VME entre os mesmos construtos foram maiores do que as correlações entre os demais fatores^(14)^.

No que se refere à consistência interna, notou-se que os coeficientes alfa de Cronbach alcançados no presente estudo foram inferiores aos encontrados em outros estudos anteriormente publicados^(5,16-18)^. Entretanto, considerando o recomendado pela literatura^(14)^, os dados obtidos demonstraram evidências de confiabilidade. Vale destacar que a presente pesquisa também utilizou a CC para mensurar a confiabilidade, pois é considerada uma medida mais adequada devido à sua menor sensibilidade com relação à quantidade de itens em cada construto^(14)^.

Além da análise fatorial, outra forma de avaliar a validade de construto foi por meio do teste de hipótese. A hipótese inicialmente formulada foi confirmada, e as relações significantes de magnitudes moderada e forte foram encontradas entre os padrões da versão brasileira da HWEAT e as três seções da versão brasileira da HSOPSC. Tais resultados demonstram que um ambiente de trabalho saudável colabora positivamente para a consolidação de uma cultura mais segura para o paciente, tal como constatado por outros autores^(19)^.

O teste de hipótese também foi utilizado na validação da versão original da ferramenta. Uma seção da HSOPSC foi utilizada para esta finalidade, e resultados similares aos da versão brasileira foram encontrados^(5)^. O teste de hipótese da versão canadense foi realizado considerando uma questão sobre a percepção dos enfermeiros em relação à qualidade do cuidado e outra sobre a segurança do cuidado. Os resultados demonstraram correlações positivas de moderada magnitude, evidenciando que ambientes de trabalho saudáveis também colaboram para a qualidade do cuidado^(15)^.

Levando em consideração os estudos que tiveram como objetivo a validação do instrumento^(5,15,16)^, foi possível notar que, nos Estados Unidos (3,58 pontos) e na Arábia Saudita (3,64 pontos), a percepção dos profissionais foi mais positiva do que a dos profissionais da presente pesquisa^(5,17)^. Entretanto, nas versões canadense (2,96 pontos) e japonesa (3,00 pontos), os profissionais demonstraram uma percepção mais negativa, quando comparados aos brasileiros^(15,16)^. Esses resultados classificaram os ambientes em “bons” e “necessitam de melhorias”, o que demonstra a possibilidade de as instituições ainda explorarem e implementarem estratégias para melhorar as condições laborais dos profissionais.

Com relação aos padrões, os dados da versão brasileira se assemelharam aos de outros estudos, principalmente com relação ao padrão reconhecimento significativo, que tem recebido a menor pontuação nas pesquisas^(5,16-18,20)^. Dados de pesquisa longitudinal, que avaliou a implementação dos padrões para um ambiente de trabalho saudável, demonstraram que tanto antes quanto após a intervenção o reconhecimento significativo recebeu a pior avaliação por parte dos profissionais^(21)^. O reconhecimento é primordial e constitui o centro dos padrões, pois sua ausência pode contribuir para que as pessoas desenvolvam sentimentos de invisibilidade, desvalorização, desmotivação, desrespeito e insatisfação^(6)^. Por isso, informar os profissionais sobre a excelência de sua *performance* é reforçar o seu valor na prestação de cuidados de qualidade aos pacientes^(17)^.

Autores reforçam algumas medidas que podem ser implementadas para que o reconhecimento significativo possa ser aprimorado, além de estratégias que envolvem o aumento de custos, como maior flexibilidade do horário de trabalho, estímulo ao desenvolvimento profissional e premiações para profissionais indicados por pacientes e colegas. O reconhecimento deve se tornar uma prioridade das lideranças, e cada conquista deve ser publicamente celebrada^(22)^.

A implementação desses padrões, inclusive de comportamentos, deve ser uma prioridade das organizações de saúde, pois permite a estabilidade da força de trabalho, a promoção de uma cultura mais segurança, a melhoria do desempenho e da produtividade dos profissionais, e a melhoria da qualidade da assistência prestada ao paciente e da viabilidade financeira das instituições^(7,17,23)^. Por isso, é recomendada pela Organização das Nações Unidas, Organização Mundial da Saúde e Organização Internacional do Trabalho como um impulso ao desenvolvimento sustentável^(4,9,10)^.

### Limitações do estudo

Como limitações desta pesquisa, podem ser citadas a constituição da amostra por conveniência, que pode levar a um viés de seleção, e a utilização de ferramenta autoadministrada, que pode sofrer interferência da desejabilidade social. A não utilização de outros instrumentos que mensuram os resultados com os profissionais, como satisfação no trabalho ou estresse ocupacional, e a realização da pesquisa em um único cenário, que pode não representar a totalidade da cultura, considerando a extensão do território brasileiro, também podem ser citados como limitadores.

### Contribuições para a área da saúde

A disponibilização da versão brasileira da HWEAT possibilitará o mapeamento e a subsequente avaliação do avanço das instituições na trajetória da implementação e consolidação de um ambiente de trabalho saudável. Essa ferramenta poderá auxiliar os gestores nos esforços e investimentos necessários para implementar programas de capacitação, uma cultura de reconhecimento, políticas de tolerância zero com relação a comportamentos de abuso e desrespeito, além de combater a tradicional lacuna de cooperação interprofissional e compreender que o atendimento adequado das necessidades dos pacientes exige um maior número de profissionais qualificados^(6)^.

Já que não são consideradas estáveis^(12)^, as seguintes ações poderão ser realizadas com o intuito de possibilitar a generalização dos resultados: pesquisas futuras concretizadas em várias instituições e que levem em consideração um processo de amostragem probabilístico; a inclusão de novos instrumentos de medidas que mensurem a relação entre ambientes de trabalho saudáveis e os desfechos observados em pacientes, equipes e organizações; o mapeamento das características dos ambientes de diferentes regiões e cenários brasileiros que possibilitem o *benchmarking*; a realização de estudos longitudinais que acompanhem o progresso da implementação de estratégias que visam fomentar um ambiente de trabalho saudável^(5,6)^; e a confirmação das propriedades de medida do instrumento.

## CONCLUSÕES

A versão brasileira da HWEAT apresentou evidências de validade e confiabilidade, o que permite sua utilização junto à equipe multiprofissional, que atua em ambiente hospitalar. A ferramenta manteve a estrutura idêntica à versão original (18 itens distribuídos em seis padrões), e o teste de hipótese demonstrou que um ambiente de trabalho saudável contribui para melhorar a cultura de segurança do paciente.

## Data Availability

Os dados de pesquisa estão disponíveis em repositório: https://doi.org/10.25824/redu/Q9Q0FM.
